# Bacteriome and mycobiome and bacteriome-mycobiome interactions in head and neck squamous cell carcinoma

**DOI:** 10.18632/oncotarget.27629

**Published:** 2020-06-23

**Authors:** Elizabeth Shay, Naseer Sangwan, Roshan Padmanabhan, Scott Lundy, Brian Burkey, Charis Eng

**Affiliations:** ^1^ Genomic Medicine Institute, Lerner Research Institute, Cleveland Clinic, Cleveland, Ohio, USA; ^2^ Cleveland Clinic Lerner College of Medicine, Case Western Reserve University School of Medicine, Cleveland, Ohio, USA; ^3^ Center for Microbiome and Human Health, Lerner Research Institute, Cleveland Clinic, Cleveland, Ohio, USA; ^4^ Glickman Urological and Kidney Institute, Cleveland Clinic, Cleveland, Ohio, USA; ^5^ Taussig Cancer Institute, Cleveland Clinic, Cleveland, Ohio, USA; ^6^ Department of Genetics and Genome Sciences, Case Western Reserve University School of Medicine, Cleveland, Ohio, USA

**Keywords:** microbiome, bacteriome, mycobiome, head and neck squamous cell carcinoma, cancer

## Abstract

The etiology of head and neck squamous cell carcinoma (HNSCC) is not fully understood. While risk factors such as positive human papilloma virus (HPV) status, smoking and tobacco use have been identified, they do not account for all cases of the disease. We aimed to characterize the bacteriome, mycobiome and mycobiome-bacteriome interactions of oral wash in HNSCC patients and to determine if they are distinct from those of the oral wash of matched non-HNSCC patients. Oral wash samples were collected from 46 individuals with HNSCC and 46 controls for microbiome analyses. We identified three fungal phyla and eleven bacterial phyla of which Ascomycota (fungi, 72%) and Firmicutes (bacteria, 39%) were the most dominant, respectively. A number of organisms were identified as being differentially abundant between oral wash samples from patients with HNSCC and oral wash samples from those without HNSCC. Of note, strains of *Candida albicans* and *Rothia mucilaginosa* were differentially abundant and *Schizophyllum commune* was depleted in those with HNSCC compared to oral wash from those without HNSCC. Our results suggest that the oral cavity of HNSCC patients harbors unique differences in the mycobiome, bacteriome, and microbiome interactions when compared to those of control patients.

## INTRODUCTION

Head and neck squamous cell carcinoma (HNSCC) refers to cancer arising from the squamous epithelium of the oral cavity, pharynx, nasopharynx, and larynx. This constellation of diseases results in greater than 300,000 deaths annually, and those that do survive often suffer from significantly impaired quality of life [1, 2]. Cigarette smoking, tobacco use, and positive human papillomavirus (HPV) status are well-known risk factors for HNSCC [3]. Approximately, three-quarters of HNSCC cases can be attributed to cigarette smoking and tobacco use [4, 5]. The percentage of HNSCC attributable to HPV continues to increase and has been estimated to be as high as 39.8% at some sites [6–8]. Betel quid chewing, which is popular in India and other Asian countries, has also been shown to increase the risk of HNSCC (mainly oral cancer) [9]. Occupational exposure to substances such as wood dust, coal dust, welding fumes, asbestos and formaldehyde has been reported to promote the development of HNSCC [10]. HNSCC can also be the result of germline mutations, with Fanconi anemia being the most well-known example [11, 12]. Patients with Fanconi anemia, a heritable syndrome characterized by genomic instability, develop HNSCC at higher incidences and younger ages than the general population [13].

However, not all patients with these risk factors develop HNSCC, and some patients with HSNCC lack these risk factors. Importantly, the diagnosis of HNSCC is often delayed due to non-specific symptoms and lack of an established screening tool [14]. There is, therefore, a need to identify additional risk factors to better predict which patients, particularly among those at high risk, will develop HNSCC.

The microbiome is one important factor that may play a role in carcinogenesis, with the relationship between *Helicobacter pylori* and gastric adenocarcinoma serving as a well-established example [15]. The contribution of the microbiome to HNSCC pathogenesis, however, has not been fully explored. Dysbiosis, or alterations in the composition of microbial communities, has previously been implicated in periodontal disease [16, 17]. This is noteworthy, as the association between chronic periodontitis and HNSCC thus implies a role for dysbiosis in these cancers [18, 19]. More direct associations between HNSCC and dysbiosis have also been found. Our pilot study found evidence of epigenetic changes in HNSCC genes that were associated with certain microbial sub-populations [20]. We extended this work by demonstrating the relative depletion of certain bacterial communities in paired tumor (HNSCC) versus normal oral epithelium samples, a finding that was correlated with the extent of disease [21]. These findings highlight the association of oral dysbiosis with HNSCC.

The oral microbiome contains not only bacterial communities (bacteriome) but also fungal communities comprising the oral mycobiome [22]. Fungal communities have the potential not only to independently influence the environment of the oral cavity, but also to interact with oral bacterial communities. Recently, our group found differences in bacteriome and mycobiome correlations in oral tongue cancer (a type of HNSCC not commonly associated with HPV) compared to normal oral epithelial tissue [23]. Bacteriome-mycobiome correlations (i. e., cross-talk between the communities that is biologically relevant) from oral wash specimens have been less well characterized. Compared to that of tissue biopsies, the procedure to obtain oral wash specimens is rapid, inexpensive, and non-invasive. Determining bacteriome and mycobiome profiles as well as their correlations within oral wash samples could facilitate the development of a potential screening and high-risk surveillance tool.

We, therefore, sought to identify and characterize differences in the bacteriome and mycobiome profiles of patients with HNSCC versus healthy cancer-free patients, using oral wash as template biospecimen. To accomplish this, we performed 16S rRNA and ITS gene sequencing on oral washes from HNSCC patients and matched healthy individuals, followed by bioinformatics analysis.

## RESULTS

### Participant characteristics

We used available oral wash DNA from 46 HNSCC participants and 46 matched control participants in this study (Table 1).

**Table 1:** Participant characteristics

	Cancer (46)	Normal (46)
Age (years)	60 ± 13	59 ± 12
Male	31 (67.4%)	31(67.4%)
Race		
White	44 (95.7%)	44 (95.7%)
Black	2 (4.4%)	2 (4.4%)
Overall Stage		
Stage I-II	17 (37.0%)	
Stage III-IV	27 (58.7%)	
Unknown	2 (4.4%)	
Smoking History		
Current	3 (6.6%)	1 (2.2%)
Never	21 (45.7%)	41 (89.1)
Past	22 (47.8%)	4 (8.7%)
Alcohol Use		
Yes	27 (41.3)	34 (73.9%)
No	19 (58.7%)	10 (21.7%)
Unknown	0 (0%)	2 (4.3%)
Site		
Floor of Mouth (FOM)	2 (4.4%)	
Larynx	7 (15.2%)	
Oral cavity	3 (6.5%)	
Other	4 (8.7%)	
Pharynx/Hypopharynx	1 (2.17%)	
Tongue	22 (47.8%)	
Tonsil	7 (15.2%)	
HPV Status		
Positive	11 (23.9%)	
Negative	1 (2.2%)	
Missing	34 (74.0%)	

### Composition of the bacteriome and mycobiome of oral wash

Of the 92 samples sent for sequencing, 79 had a sufficient number of reads for mycobiome analysis. We identified three fungal phyla in the oral wash samples. Ascomycota was the predominant phylum (72.0%), followed by Basidiomycota (27.3%) and Mucoromycota (0.7%).

Of the DNA from the 92 original oral wash samples sent for sequencing, 85 had sufficient reads for bacteriome analysis. Eleven bacterial phyla were identified. Firmicutes was the most dominant (39.2%), followed by Bacteroidetes (23.2%), Actinobacteria (15.4%), Proteobacteria (9.7%), and Fusobacteria (8.0%). Other phyla present in marginal amounts were Spirochaetes (1.8%), Saccharibacteria (TM7) (1.8%), Synergistetes (0.6%), Absconditabacteria (SR1) (0.3%), Gracilibacteria (0.03%) and Chloroflexi (0.01%).

**Figure 1:**
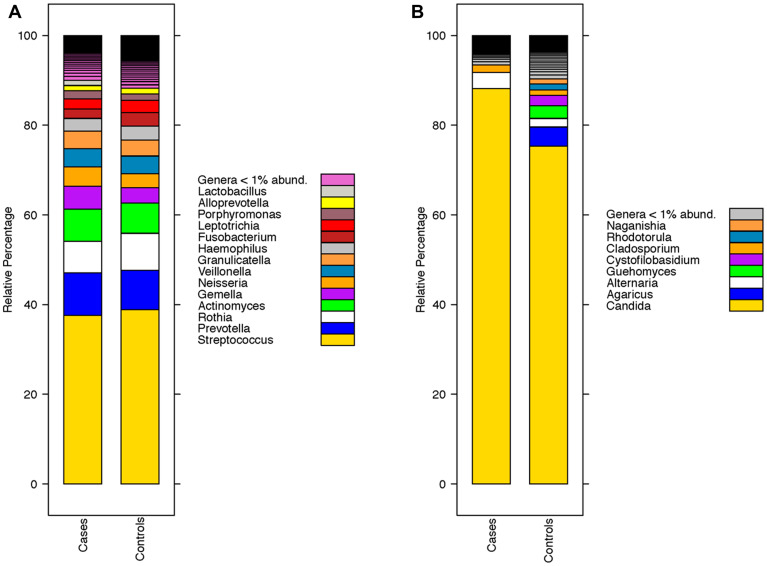
Taxonomic composition of the oral wash microbiome of head and neck squamous cell carcinoma (HNSCC) at the genus level. (**A**) Composition of the oral wash bacteriome (genus level) of cases (HNSCC patients) versus controls. (**B**) Composition of the oral wash mycobiome (genus level) of cases versus controls.

Taxonomic composition (genus level) for both mycobiome and bacteriome stratified by disease status is summarized below (Figure 1).

### Diversity of the oral bacteriome and mycobiome of HNSCC versus control patients

**Figure 2:**
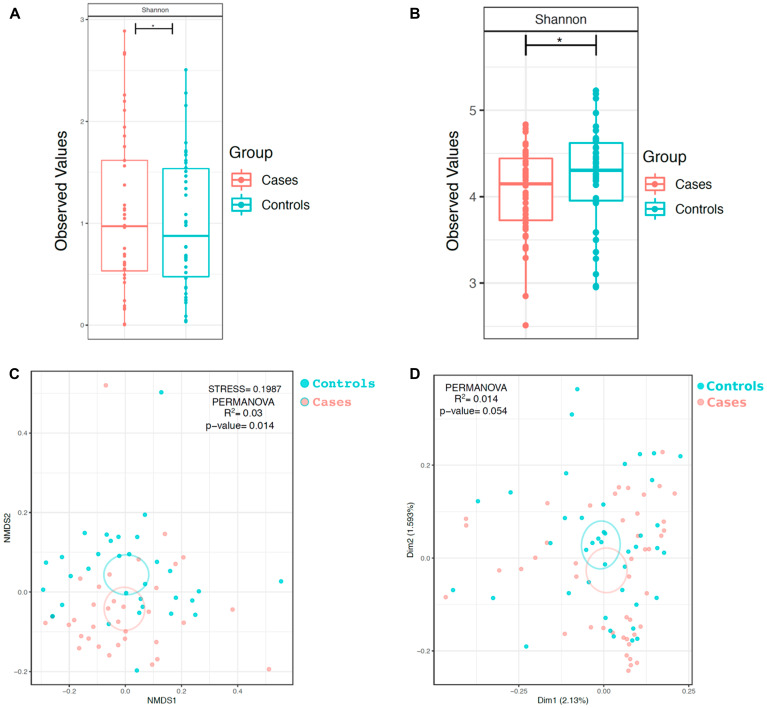
α and β-diversity of bacterial and fungal communities in HNSCC participant versus control participant oral wash. α diversity of the (**A**) bacteriome and (**B**) mycobiome based on cancer status. β diversity of the (**C**) bacteriome and (**D**) mycobiome based on cancer status. ^*^ *p* <0.05, ^**^ *p <* 0.01, ^***^ *p* <0.001

In order to determine if within-sample diversity (α-diversity) of each group differed, we calculated the Shannon diversity index (which takes into account both evenness and richness of communities) of the samples in each group. Evaluation of the bacteriome revealed that the α-diversity of case oral wash, as measured by the Shannon diversity index, was reduced relative to control oral wash (Shannon *p <* 0.05, Figure 2A). When evaluating the mycobiome, the α-diversity (Shannon diversity index) of HNSCC oral wash was noted to be reduced relative to that of control oral wash (*p <* 0.05, Figure 2B). Comparison of α-diversity by site demonstrated statistically significant differences between sub-groups (Supplementary Figure 1).

Bray-Curtis dissimilarity index was used to determine differences in the taxonomic composition (bacterial) between the case and control groups (β-diversity) (Figure 2C). Oral wash samples clustered similarly and there was no statistically significant difference between the groups (*p =* 0.054). Similar analysis was undertaken to compare how cancer and control groups differed based on fungal taxonomic composition (Figure 2D). Oral wash samples in both cohorts clustered separately by disease status (*p =* 0.01). β-diversity comparisons of the groups by site of cancer showed that samples clustered separately by site for both the mycobiome and bacteriome (Supplementary Figure 1). Samples also clustered separately when analyzing ethanol use and smoking history. (Supplementary Figure 2).

### Differential abundance analysis

**Figure 3:**
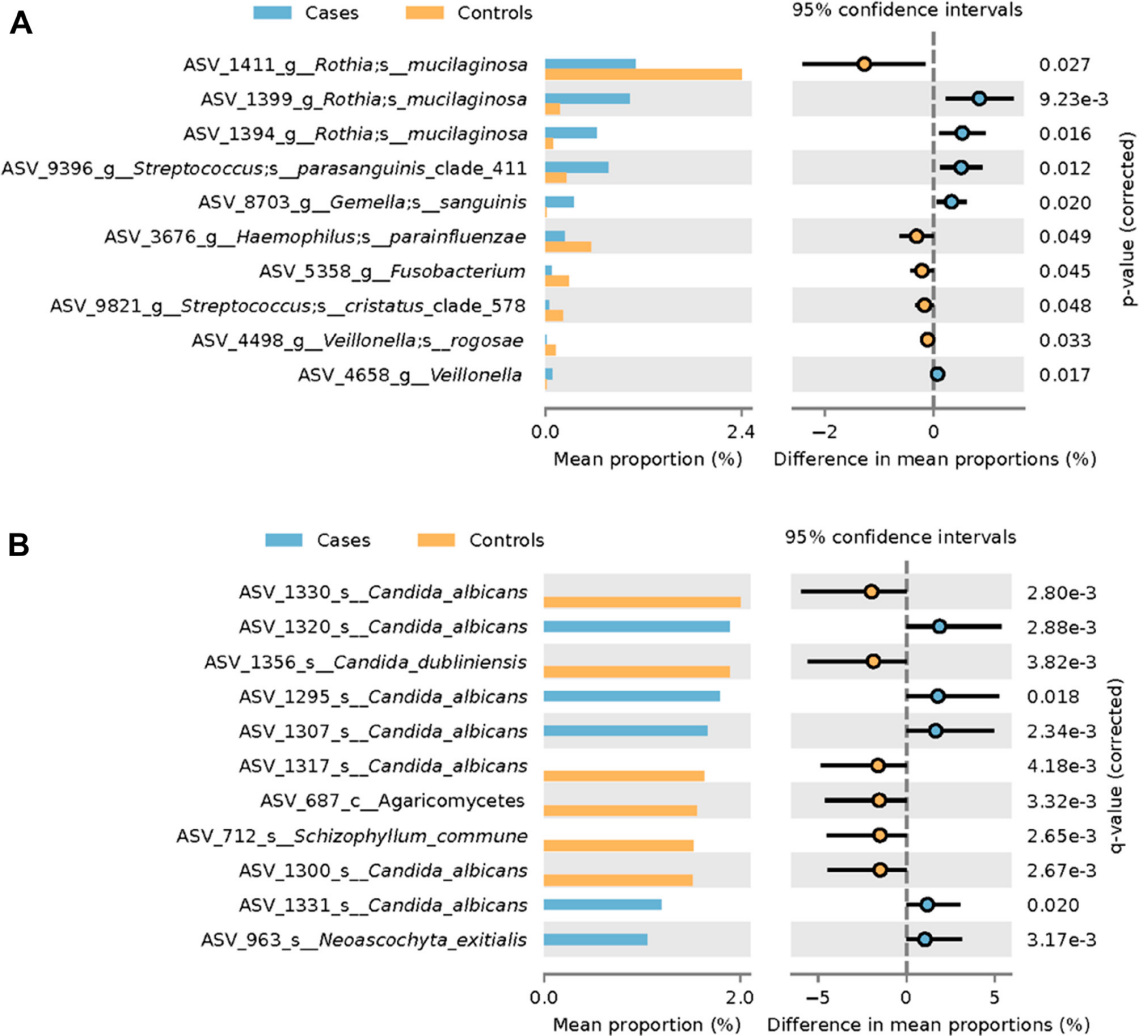
Differential abundance analysis of HNSCC participant versus control participant oral wash. Differential abundance analysis of (**A**) fungi and (**B**) bacteria in HNSCC participant versus control participant oral wash.

Analysis (taking into account smoking and ethanol use history) was conducted to determine which organisms were differentially abundant when comparing oral wash obtained from case versus control participants (Figure 3). Specific strains of *Candida albicans* were found to be both overrepresented and underrepresented in oral wash samples from cancer patients relative to those from non-cancer controls (Figure 3A). *Candida dubliniensis*, *Schizophyllum commune* and an organism from the class *Agaricomycetes* were found to be overrepresented in controls relative to cases. By contrast, one strain of *Neoascochyta exitialis* was found to be underrepresented in oral wash from cases relative to that of controls.

Differential abundance analysis of bacteria in oral wash revealed two strains of *Rothia mucilaginosa* that were overrepresented in the oral wash of cases relative to that of controls (Figure 3B). Additionally, one strain of *Rothia mucilaginosa* was underrepresented in case oral wash versus control oral wash. Strains of *Streptococcus parasanguinis*, G*emella sanguinis* and an organism from the genera *Veillonella* were overrepresented in case oral wash versus control oral wash. Strains of *Haemophilus parainfluenzae, Streptococcus cristatus, Veillonella rogosae* and an organism from the genera *Fusobacterium* were underrepresented in cancer oral wash relative to control oral wash.

**Figure 4:**
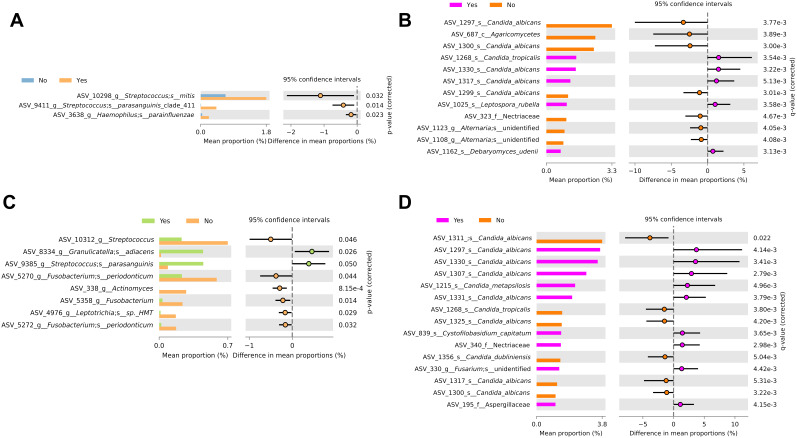
Differential abundance analysis by ethanol use and smoking history. Differential abundance analysis of the (**A**) bacteriome and (**B**) mycobiome based on ethanol use. Differential abundance analysis of the (**C**) bacteriome and (**D**) mycobiome based on smoking history.

Differential abundance analysis comparing ethanol use and smoking history was performed for the bacteriome and mycobiome (Figure 4A–4D).

### Exploring microbe-microbe interactions with network analysis

**Figure 5:**
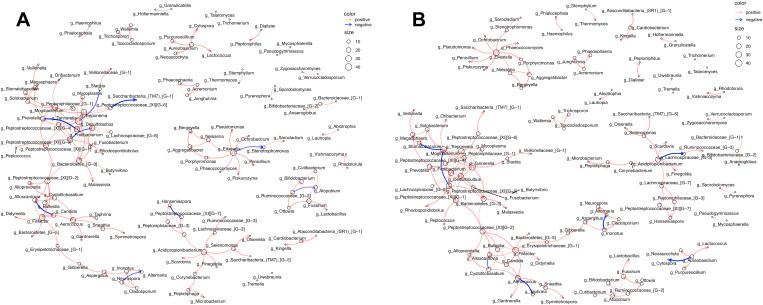
Network analysis depicting intra-kingdom and inter-kingdom correlations. Correlations within (**A**) case oral wash and (**B**) control oral wash.

Network analysis revealed the presence of intra-and inter kingdom interactions within the oral wash of case and control participants (Figure 5). Each network comprised of multiple clusters containing predominantly bacterial organisms and fewer fungal organisms. The two largest clusters in both case and control networks were connected and contained many of the same organisms. Interestingly, the interaction between Peptostreptococcaceae and Saccharibacteria was negative in case oral wash but positive in control oral wash. Similarly, Prevotella and Freitibacterium correlated negatively in case oral wash, but positively in control oral wash. Additionally, Candida correlated negatively with Alloscardovia in case oral wash, but positively in control oral wash.

## DISCUSSION

Previous studies have shown associations between the oral microbiome and HNSCC. These studies, have, however, primarily focused on the bacteriome. This study is one of a few that have explored the mycobiome profiles in HNSCC from oral wash [23–25]. Here, we show that individual bacterial and fungal organisms display association with HNSCC status and that these microorganisms interact with each other.

The oral mycobiomes of both HNSCC and healthy participants were dominated by the genus *Candida* (Figure 1). This observation has been reported in several studies of the oral mycobiome in healthy and diseased states [22, 26, 27]. We also observed that varying strains of *Candida albicans* were enriched in both diseased and healthy participants. Oral candidiasis has been strongly associated with the risk of development of various malignancies, including those of the head and neck [28]. Perera et al. similarly noted that *Candida albicans* was elevated in oral squamous cell carcinoma tissue compared to benign tissue (intra-oral fibro-epithelial polyps) [24]. Vesty et al. also reported an abundance of *Candida albicans* in the saliva of HNSCC patients that correlated with the abundance of the inflammatory cytokines IL-1β and IL-8 [25]. This finding implies a role for *Candida albicans* in inducing inflammation, perhaps via hypermethylation of tumor suppressors [25, 29]. That we observed enrichment of *C. albicans* in both healthy and diseased participants does not negate evidence that the organism may be involved in HNSCC carcinogenesis. It is known, for example, that the association of *H. Pylori* with gastric cancer is driven by specific pathogenic strains [30–32]. It is, therefore, plausible that our study identified both pathogenic and non-pathogenic *C. albicans* strains. Further research is needed to characterize the specific *C. albicans* strains associated with HNSCC status. Accomplishing this goal could increase the specificity of a microbiome-based oral wash screening tool for HNSCC. A second fungi of interest, *Schizophyllum commune,* was enriched in control oral wash. The genera *Schizophyllum* is a member of the phylum Basidiomycota and has been previously reported as a member of the oral mycobiome [33]. *Schizophyllum commune* is known to produce the polysaccharide compound schizophylan [34]. Schizophylan has anti-tumor properties in-vitro and showed some promise in treating cancer (including HNSCC) in studies conducted in Japan in the 1980s [34–37]. The enrichment of this organism in control participants, therefore, supports a role for continued investigation into Schizophylan’s anti-cancer properties.

We next explored the bacteriome of the samples. A number of the bacterial organisms observed to be overrepresented in our HNSCC cohort have previously been reported to be associated with HNSCC. Species from the genus *Gemella,* including *Gemella sanguinis,* have been found to be increased in oral squamous cell carcinoma tissue [38, 39]. Interestingly, *Gemella* bacteremia has also been associated with subsequent colorectal carcinoma diagnosis [40]. *Streptococcus parasanguinis,* although considered part of a healthy oral microbiome, has been reported to be associated with tumor site in oral squamous cell carcinomas [38]. Conversely, *Streptococcus cristatus,* an oral commensal, was depleted in HNSCC versus control oral wash. *Streptococcus cristatus* co-aggregates with *Fusobacterium nucleatum* and Zhang et al. reported that *Streptococcus cristatus* could attenuate inflammation induced by *Fusobacterium nucleatum* [41, 42]. The relative depletion of *Streptococcus cristatus* in HNSCC participant oral wash versus control wash could, therefore, mean the loss of a commensal that reduces cancer-promoting inflammation. Given the association of *Fusobacterium nucleatum* with cancer status, it was perhaps surprising that we noted the genera *Fusobacterium* to be enriched in case oral wash. In fact, other species from the genera *Fusobacterium,* such as *Fusobacterium periodonticum*, have, in some instances, been reported to be depleted in HNSCC [43]. Consistent with our results, *Rothia* has also been reported as both enriched and depleted in HNSCC tissue and biospecimen versus that of control [23, 44, 45].

It is thus apparent that there are distinctions in the relative abundance of bacterial and fungal organisms between the groups. These data provide a basis for a potential screening tool for HNSCC based on bacteriome and mycobiome differences. Interestingly, none of the aforementioned bacterial organisms was found to be associated with an increased risk of HNSCC in a large case-control study nested within two prospective cohort studies that assessed for incident HNSCC risk [46]. It is important to highlight that Hayes et al. profiled pre-diagnosis oral wash samples; these discrepant findings could reflect differences between the microbiome of individuals predisposed to developing HNSCC and that of individuals with HNSCC pathogenesis-related changes. This type of contrasting observations can help to delineate causation, progression (i. e., pathogenesis) and post-cancer microbiome changes, potentially unrelated to initiation. Large prospective longitudinal studies are needed to further outline the role of bacterial and fungal organisms in the etiology and pathogenesis of HNSCC. Although it may be challenging to establish causality, our findings offer an opportunity to further efforts to use oral wash as a screening tool for HNSCC.

The organisms that comprise our microbiome interact with each other. Correlations between organisms present in HNSCC tumor samples and bio specimen have been previously noted. In this study, we found inter and intra-kingdom correlations within oral wash. Although the composition of the clusters within networks appeared largely similar between case and control oral wash, there were some interactions that differed. A positive relationship between two organisms could suggest that they occupy similar niches or even that they share a symbiotic relationship. A negative relationship, by contrast could point to two organisms that either compete against each other through varying means. That we noted multiple interactions that were opposing when considering case oral wash versus control oral wash suggests not only changes in the composition of the microbiome, but also in how members of the microbiome interact with each other in HNSCC patients. The relationship between *Alloscardovia* and *Candida,* for example, was negative, in case oral wash but positive in control oral wash. Such shifts could signal the presence of HNSCC in an oral wash based screening tool.

## MATERIALS AND METHODS

### Participant enrollment and oral rinse collection

Approval was obtained from the Institutional Review Board (IRB) for Human Subjects Protection at the Cleveland Clinic. Consent was obtained from patients with HNSCC and normal healthy individuals without malignant dental or airway issues. Participants were asked to provide basic demographic information. Oral rinse was collected prior to surgical treatment, chemotherapy and radiation with the exception of two patients, (one of whom had previously had radiation and surgical treatment, and another who had previously only received radiation). Samples were only collected from participants who had not eaten or drunk anything but water for the previous 30 minutes. Participants were asked to rinse their mouths with normal saline for one minute, which was subsequently spit into a collection cup. This process was repeated once. Oral rinse was processed for storage at the Genomic Medicine Biorepository (GMB) within two hours of collection time. Participants were matched by age, sex and ancestry.

### DNA extraction

Previously extracted and stored DNA was used. The cell pellet was resuspended in 650 ul of lysis buffer, and transferred to TissueLyzer II (Qiagen, Venlo, Netherlands). The TissueLyzer II was set for two rounds of 30Hz for 10 minutes. The plates were then centrifuged for 9 minutes at 3000 g before adding 150 ul of inhibitor removal solution to a new plate along with supernatant from previous plate. The plate was vortexed for 5 seconds, incubated at 4° C for 5 minutes and then centrifuged again for 9 minutes at 3000 g. The supernatatant was transferred to a new plate and centrifuged again. The previous step was repeated once. Beads were then added (870 ul of a 2 ml bead solution with 85 ml binding solution), the solution mixed and placed on the magnet until solution was clear. The supernatant was discarded and the beads were washed twice using 500 ul of wash buffer. After a final wash step using 500 ul of wash buffer, the supernatant was discarded and 100 ul of water added. The solution was mixed for 10 minutes before placing plate on magnet and transferring 100 ul of extracted DNA for storage.

After undergoing processing using the TissueLyser II (Qiagen), a subset of nineteen samples were instead processed with the Masterpure Yeast DNA purification kit according to manufacturer instructions (Epicentre, Madison, WI, USA). The QIAmp DNA mini kit (Qiagen) was used to complete the DNA purifications. All beads, tubes, and nonenzymatic reagants were treated with UV light for at least 30 minutes prior to use. Reagent controls were confirmed by 16S rRNA gene PCR to be absent of contaminating bacteria. Differences in extraction technique were included as a covariate in the analysis.

### Amplification and sequencing

PCR amplification of the V1-V2 region of the 16S rRNA gene and regions of the ITS rRNA gene was accomplished using the QIAseq 16S/ITS Region Panels (Qiagen). PCR was performed with the following conditions for amplification of ITS rRNA gene: 95C for 5 mins, followed by 20 cycles of 95C for 30 seconds, 50C for 30 seconds, 72C for 2 mins, and an extension of 72C for 7 mins. PCR amplification of the V1-V2 hypervariable regions of the 16S rRNA gene was performed under the following conditions: 95C for 5 mins, followed by 16 cycles of 95C for 30 seconds, 50C for 30 seconds, 72C for 2 mins, and an extension of 72C for 7 mins. Two rounds of bead purification followed PCR amplification. Adapters were added to the ends of amplicons and libraries were generated using the QIAseq 16S/ITS index kit (Qiagen). The PCR conditions for the index PCR reaction were the same for bacterial and fungal samples and were as follows: 95C for 2 mins, followed by 19 cycles of 95C for 30 seconds, 60C for 30 seconds, 72C for 2 mins, and an extension of 72C for 7 mins. One round of bead purification of libraries was then performed. Quantification of final libraries was accomplished using the Qubit Fluorometer dsDNA broad range assay. Verification of the appropriate fragment size for each sample was completed with Invitrogen e-Gel. Libraries were pooled in equamolar volumes and the final library pool quantified with qPCR (NEBNext Illumina Library Quant kit). High-throughput sequencing was completed at the Case Western Reserve University Genomics Core using the Illumina MiSeq v3 paired-end flow cell after dilution and denaturation of the pool.

### Bioinformatic analysis

The quality of the sequences was assessed using FastQC and MultiQC. Paired end sequences were imported into QIIME 2 (2018.8) using the Casava 1.8 paired-end demultiplexed fastq format [47]. The Divisive Amplicon Denoising Algorithm (DADA) 2 pipeline, within QIIME 2, was used to trim sequences, conduct dereplication, detect and filter chimeric sequences, and merge paired ends [48]. DADA2 uses an algorithm to model and correct amplicon errors and is more reliable than OTU construction methods. The first 20 bp were trimmed from the beginning of bacterial sequences and reads were truncated at 245 bp. Fungal sequences were trimmed by 20 bp and were truncated at 250 bp. A feature table (the QIIME 2 equivalent of an OTU table), phylogenetic tree and taxonomy file were constructed within QIIME 2. Bacterial sequences were classified against HOMD v 15.1, and fungal sequences against UNITE (Version 18.11.2018) after classifier training within QIIME 2 [49, 50]. The output of the dada2 pipeline (feature table of amplicon sequence variants (an ASV table)) was processed for alpha and beta diversity analysis using *phyloseq*, and microbiomeSeq (http://www.github.com/umerijaz/microbiomeSeq) packages in R [51]. Alpha diversity estimates were measured within group categories using estimate_richness function of the *phyloseq* package [51]. Multidimensional scaling (MDS, also known as principal coordinate analysis; PCoA) was performed using Bray-Curtis dissimilarity matrix between groups and visualized by using *ggplot2* package [52]. We assessed the statistical significance (*P* < 0.05) throughout and whenever necessary, we adjusted *P*-values for multiple comparisons according to the Benjamini and Hochberg method to control False Discovery Rate while performing multiple testing on taxa abundance according to sample categories [53]. We performed an analysis of variance (ANOVA) among sample categories while measuring the of α-diversity. Permutational multivariate analysis of variance (PERMANOVA) with 999 permutations was performed on all principal coordinates obtained during PCoA with the *ordination* function of the *microbiomeSeq* package. Linear regression (parametric test), and Wilcoxon (Non-parametric) test were performed on ASVs abundances against coprostanol levels using their base functions in R. Co-occurrence patterns were analyzed between features/taxa (bacterial and fungal taxa) using the ‘co_occurence_network’ function in the microbiomeSeq package. The following parameters were used: grouping_column = “Variable”, rhos = 0.65, method = “cor”, qval_threshold = 0.05. The resulting co-occurrence object was converted into a graph object using igraph2 and plotted using the ggraph3 package. The size and width of the nodes is proportional to the degree and correlation between the two nodes, respectively. Positive correlations between nodes are represented using the color red and negative correlations are represented using the color blue. The analysis within this study was conducted correcting for age, DNA extraction method, smoking status (tobacco use) and ethanol use.

## CONCLUSIONS

There are distinctions in the oral mycobiome and bacteriome as well as microbiome correlations in the oral cavity, reflected here by oral wash, of HNSCC patients when compared to those of healthy individuals. Corroboration of our findings, particularly in prospective longitudinal studies, could help to further research to facilitate the development of non-invasive strategies to identify high-risk patients based on their oral wash bacteriome and mycobiome profiles. Such studies would help to clarify the temporal order of dysbiosis and carcinogenesis and potentially establish causality. Additionally, we envision the use of probiotics and anti-fungals to modulate dysbiosis and therefore reduce the risk of HNSCC development and/or pathogenesis. There is already one probiotic product (BIOHM) that combines beneficial bacteria and fungi for improved digestive health [54]. Our research adds to the body of research on the microbiome of HNSCC that would inform the development of a similar product targeted to eliminate HNSCC associated dysbiosis.

## SUPPLEMENTARY MATERIALS


