# Supplementary material for: Bacteriome and mycobiome and bacteriome-mycobiome interactions in head and neck squamous cell carcinoma
Source: Oncotarget. 2020 Jun 23;11(25):2375–86. doi: 10.18632/oncotarget.27629 (PMC7321695; doi:10.18632/oncotarget.27629)
Supplement: Supplementary file 1 [file oncotarget-11-2375-s001.pdf]

Bacteriome and mycobiome and bacteriome-mycobiome interactions in head and neck squamous cell carcinoma

SUPPLEMENTARY MATERIALS

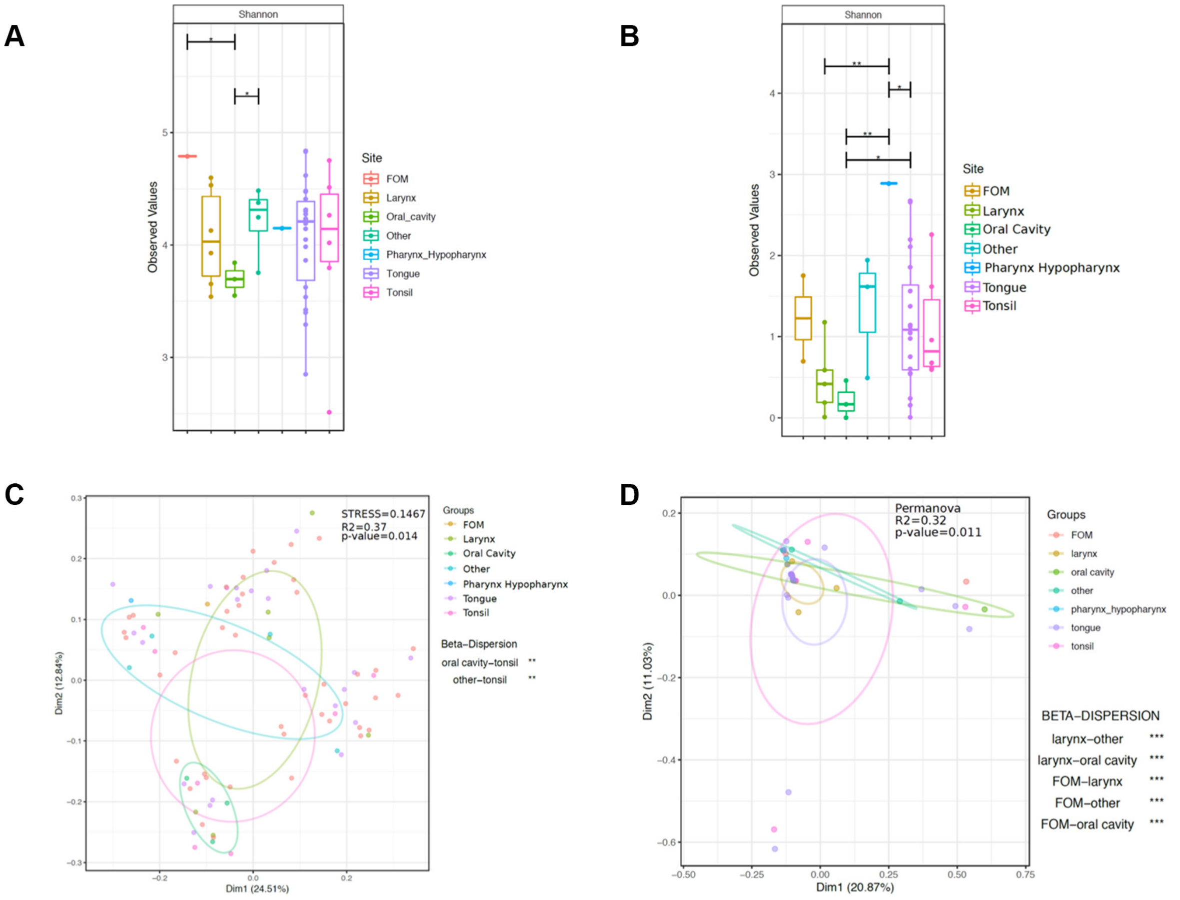

Supplementary Figure 1: α and β-diversity of bacterial and fungal communities in HNSCC participants by site of cancer. (**A**) α diversity of the bacteriome by site of cancer (**B**) α diversity of the mycobiome by site of cancer (**C**) β-diversity of the bacteriome by site of cancer (**D**) β-diversity of the mycobiome by site of cancer. $^{*}p < 0.05$, $^{**}p < 0.01$, $^{***}p < 0.001$.

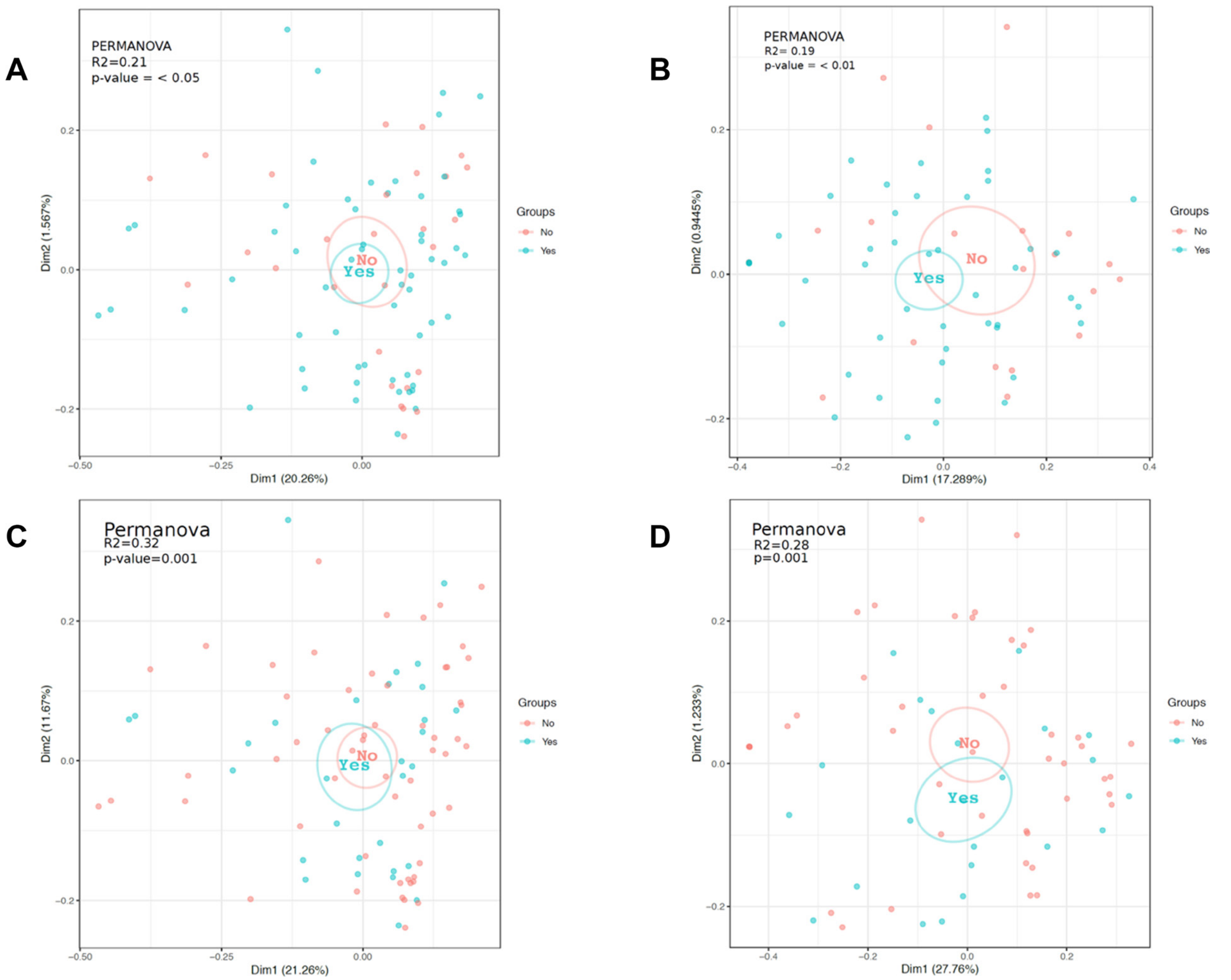

Supplementary Figure 2: β-diversity by ethanol use status and smoking history. β-diversity of the (**A**) bacteriome and (**B**) mycobiome based on ethanol use. β-diversity of the (**C**) bacteriome and (**D**) mycobiome based on smoking history.
